# Descriptive Epidemiology of Rescue-Related Fatal Drowning in Turkey

**DOI:** 10.3390/ijerph18126613

**Published:** 2021-06-19

**Authors:** Ali Işın, Adnan Turgut, Amy E. Peden

**Affiliations:** 1 Department of Coaching Education, Faculty of Sports Sciences, Akdeniz University, Antalya 07070, Turkey; isin_ali@hotmail.com; 2 Department of Physical Education and Sports, Faculty of Sports Sciences, Akdeniz University, Antalya 07070, Turkey; turgut@akdeniz.edu.tr; 3 School of Population Health, Faculty of Medicine, University of New South Wales, Sydney, NSW 2052, Australia

**Keywords:** drowning, rescue, multiple drowning incident, prevention, injury, risk, epidemiology

## Abstract

Drowning is a public-health threat and a leading cause of injury-related death. In Turkey, drowning results in 900 fatalities annually, and the rate is rising. As data on rescue-related drowning are scarce, this retrospective study explores the epidemiology of fatal drowning among rescuers in Turkey. As there are no routinely collected death registry data on drowning in Turkey, data were sourced from media reports of incidents between 2015 and 2019. Rescuer fatalities were analysed by age, sex, activity prior to rescue, location, incident day of week and season, and place of death. Statistical analyses comprised X^2^ tests of significance (*p* < 0.05) and calculation of relative risk (95% confidence interval) using fatality rates. In total, 237 bystander rescuers drowned (90% male; 35% 15–24 years). In 33% of cases, the primary drowning victim (PDV) was successfully rescued, while in 46% of cases the rescue resulted in multiple drowning fatalities (mean = 2.29; range 1–5 rescuers). Rescues were more likely to be successful in saving the PDV if undertaken at the beach/sea (X^2^ = 29.147; *p* < 0.001), while swimming (X^2^ = 12.504; *p* = 0.001), or during summer (X^2^ = 8.223; *p* = 0.029). Risk of bystander rescue-related fatal drowning was twice as high on weekdays compared to on weekends (RR = 2.04; 95%CI: 1.56–2.67). While bystanders play an important role in reducing drowning, undertaking a rescue is not without risk and can lead to multiple drowning incidents. Training in rescue and resuscitation skills (especially the prioritization of non-contact rescues) coupled with increasing awareness of drowning risk, are risk-reduction strategies which should be explored in Turkey.

## 1. Introduction

Drowning is a globally neglected threat to public health, resulting in 295,000 deaths worldwide each year [1]. However, this estimate excludes transportation and disaster-related drowning fatalities [2], meaning the true burden is likely to be significantly higher. In Turkey, the unintentional fatal drowning rate is increasing; it rose from 0.89 per 100,000 population [3] between 2005 and 2011 to a current rate of 1.16 per 100,000 population [4].

Drowning is defined as a process, with outcomes ranging from death to survival with no ongoing injury [5]. Often those who experience non-fatal drowning are rescued at some stage during the drowning process, either by trained or volunteer lifesavers and lifeguards, or bystanders [6,7,8]. Surfers have also emerged as a group performing rescues in coastal environments [9]. Performing a rescue is not without risk and a small body of literature has emerged exploring the rescuer who drowns (the aquatic victim instead of rescuer [AVIR]) phenomena [8,10,11,12]. Such cases are often altruistic in nature, with rescues typically involving a child or loved one [10]. However, there is often a mismatch between willingness to perform an aquatic rescue and the skills needed to do so, particularly in open water, where most drowning incidents occur [13]. This indicates the need to provide training to potential bystander rescuers on rescue safety, something many report as never having been taught [14].

Drowning incidents which result in multiple fatalities (i.e., where both the rescuer and rescue drown), are a significant issue [11], contributing to the rising fatal drowning rate in Turkey [4]. As such, this present study aimed to investigate cases of the fatal drowning of bystander rescuers in Turkey between 2015–2019. In addition to identifying a range of risk factors, this study also explored multiple fatality events which occur during fatal bystander rescues to inform prevention efforts and curb Turkey’s rising drowning toll.

## 2. Materials and Methods

This study takes a public health approach [15] to the issue of bystander rescue-related fatal unintentional drowning in Turkey. We do this through the collation of data to define the issue and conducting analysis to identify risk factors, both of which are used to provide evidence-informed recommendations for reducing such drowning incidents in Turkey.

### 2.1. Study Design

This retrospective study was undertaken exploring the fatal drowning of bystander rescuers which occurred in Turkey between 1 January 2015, and 31 December 2019. Bystander rescuers were defined as those who rescued someone from drowning while not acting in an official capacity (i.e., those who drowned while conducting a rescue while working as lifesavers or lifeguards were not included in this study). All multiple drowning incidents investigated in this study include bystander rescuers (i.e., incidents where more than one rescuer drowned). Thus, any drowning cases consisting of more than one victim without the drowning fatality of a bystander rescuer were not included in this study.

### 2.2. Data Collection and Coding

There are a lack of official statistics on drowning in Turkey [11]. Currently, the Turkish Statistical Institute (TurkStat) publishes annual data on causes of death (including external causes of injury and poisoning); however, such data are limited in their disaggregation and do not contain deaths by injury mechanism (such as drowning) [16]. In lieu of official data, fatal drowning incidents are collated using media reports [3,4,17]. This method has been used in many other countries in lieu of, or to supplement, official drowning statistics [18,19,20,21].

All unintentional drowning-related data were collected from online media reports via Google News [22]. Daily searches were run using regular and advanced search functions limiting region to Turkey and language to Turkish. A daily Google News digest email was also received to collate relevant cases. Search terms used were “boğulma” (drowning), “ölümcül boğulma” (fatal drowning), “kurtarma girişimi sırasında boğulma” (drowned during rescue attempt), and “kurtarıldı ve boğuldu” (rescued and drowned). Only news items published between 1 January 2015, and 31 December 2019 about incidents during the same period were included for analyses. Only unintentional fatal drowning events associated with rescues were evaluated. If a case was reported by more than one news source, these cases were evaluated as a single case. Where data reported were inconsistent, the name of the victim was searched across multiple reports and data confirmed.

The following data were extracted for each case and used in analyses: age, gender, activity prior to drowning, location of drowning incident, season of drowning incident, place of death and day of the week. The success of the bystander rescuer in successful rescuing the primary drowning victim was assessed and in cases of multiple drowning incidents, the number of fatalities were recorded.

The seasons of drowning incidents were classified as follows: Winter: December to February; Spring: March to May; Summer: June to August; Autumn: September to November. Place of death was coded into ‘at the scene’ or ‘hospital’. The days of the week were coded into two groups as weekdays (Monday–Friday) and weekends (Saturday and Sunday). The activity being undertaken by the rescuer immediately prior to performing the rescue was coded as swimming, non-water related recreation, having a picnic, occupational (i.e., farmer, technician, self-employed, etc.) recreational fishing, boating and other. The place where the drowning occurred were coded as stream/river/creek, beach/sea, dam, lake/pond, irrigation channel, pool, and water hole. The two cases involving riverine floods were coded to stream/river/creek. Age groups were coded as 0–4 years, 5–14 years, 15–24 years, 25–34 years, 35–44 years, 45–54 years, 55 years and older.

### 2.3. Data Analysis

Descriptive data of the rescuers who drowned while attempting a rescue were presented as frequency (f) and percentage (%). Chi square tests of significance were calculated using a Fisher’s Exact Test (*p* < 0.05). Population data were retrieved from the Turkish Statistical Institute to calculate the mortality rate (per 100,000 population) [23]. Relative risk (RR) with a 95% confidence interval (95%CI) was calculated for gender, age group, day of the week and seasons. In the calculation of RR and CI, the group with the lowest number of cases were used as the reference [4].

### 2.4. Ethics Approval

This study received ethics approval from the University of New South Wales Human Research Ethics Committee (HC210244).

## 3. Results

**Figure 1:**
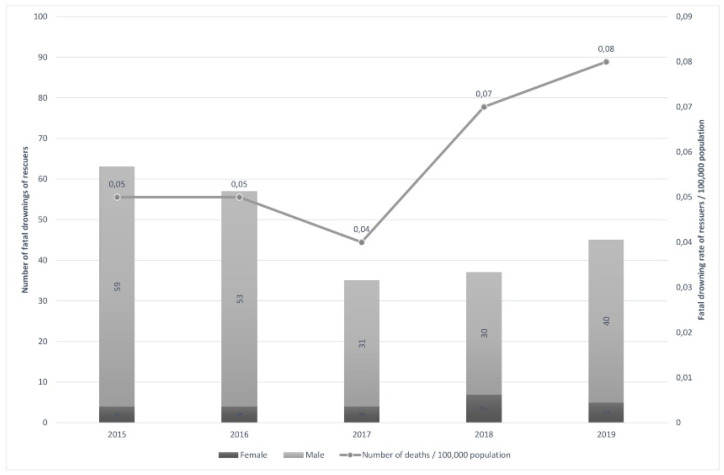
Frequency of unintentional bystander rescue-related fatal drowning and crude rate per 100,000 population by year of incident and sex; Turkey, 2015–2019.

Across the study period, 237 bystander rescuers drowned. Annual mortality rates varied from a low of 0.04/100,000 people in 2017 to a high of 0.08 in 2019 (Figure 1). Males accounted for 89.9% of all bystander rescue-related drowning fatalities. The age group most commonly represent among bystander rescuer drowning fatalities was the 15–24 years age group (35.0%). This was followed by the 25–34 years age group (20.7%) and the 5–14 years age group (14.3%) (Table 1).

Bystander rescue-related fatal drownings were more likely to occur on weekdays (67.1%) (Table 1). A slightly higher proportion of female bystander rescue related fatal drownings occurred on weekends (33.3%) as opposed to males (32.9%), although this difference was not statistically significant (Table 2).

The vast majority of drowning fatalities occurred at the scene (89.9%), with just 10.1% of bystander rescuers dying in hospital (Table 1). There were no statistically significant differences in place of death by sex of rescuer (Table 2).

Summer (63.7%), followed by Spring (21.5%) are the seasons with the highest proportions of bystander rescue-related drowning fatalities. A slightly higher proportion of females drowned in bystander-rescue-related incidents in Spring (25.0%) compared to males (21.1%); however, there was no statistically significant difference by sex of rescuer and season of incident (Table 2).

Swimming (29.1%) was the leading activity in fatal drownings of bystander rescuers, followed by non-water related recreation (23.2%) and having a picnic (18.6%). When activity prior to bystander rescue-related drowning was explored by sex of the rescuer, females were significantly more likely to fatally drown while conducting a bystander rescue while having a picnic (X^2^ = 6.333; *p* = 0.023). Stream/River/Creek (34.7%) was the leading location for bystander rescue-related drowning fatalities, followed by Beach/Sea (24.9%) and Dam (12.7%) (Table 1). There were no significant differences in bystander rescue drowning fatalities by sex and location of drowning incident (Table 2).

When exploring rescue attempts by success of rescuing the person who was drowning, in 77 cases (32.5%) the primary drowning victim was rescued. In 54.4% (*n* = 129) of cases there was one bystander rescuer who drowned. In almost half of all cases (45.5%; *n* = 108) the bystander rescue resulted in multiple rescuers drowning (mean = 2.29; range 1–5 rescuers) (Table 3).

Rescues were significantly more likely to be successful in saving the primary drowning victim if they occurred at the beach or sea (X^2^ = 29.147; *p* < 0.001), while swimming (X^2^ = 12.504; *p* = 0.001), or during the summer months (X^2^ = 8.223; *p* = 0.004). Bystander rescues were more likely to be unsuccessful in saving the primary drowning victim if they occurred during non-water related activities (X^2^ = 5.093; *p* < 0.032) or during Spring (X^2^ = 4.916; *p* < 0.029) (Table 1).

When compared to females, male bystander rescuers drowned during rescue attempts almost nine-times (RR = 8.81; 95%CI: 5.78–13.44) more frequently. Risk of fatally drowning while undertaking a bystander rescue was highest among those 15–24 years old, 82-times higher than the risk for 0–4-year-old individuals (RR = 82.21; 95%CI: 11.44–253.04). The risk of death on weekdays was twice as high (RR = 2.04; 95%CI: 1.56–2.67) compared to the weekend. Compared to winter, the highest risk of drowning was in the summer (RR = 13.73; 95%CI: 7.45–25.30), followed by spring (RR = 4.64; 95%CI: 2.42–8.90) and autumn (RR = 2.18; 95%CI: 1.07–4.45) (Table 4).

## 4. Discussion

Drowning is a leading cause of preventable death globally and in Turkey [1]. Although many measures have been recommended to prevent such deaths [24], drowning fatalities in Turkey are increasing [4]. While bystander rescuers play an important role in preventing drowning, especially in unpatrolled locations or in countries without lifeguarding or lifesaving services, this is not without risk [8,10,11,12]. This study reports the demographics and risk factors associated with fatal drowning of bystanders during rescues in Turkey and found that bystander rescue-related drowning fatalities have doubled since 2017, contributing to the rising fatal drowning toll in Turkey [4].

In the present study, male rescuers were eight-times more likely to drown while performing a bystander rescue than females. Similarly, the overrepresentation of males in rescue-related drowning has previously been identified [8,11,20]. Gender is among one of the most important risk factors for drowning, and it is known that males have a higher risk of drowning than females [17,25,26,27]. When the underlying reasons are examined, it has been reported that males are exposed to water more than females, and they participate in water-based recreational activities more frequently, spend longer in deeper water and are more likely to enter the water under the influence of alcohol or refuse to wear a lifejacket [4,28,29,30,31,32]. Increased exposure coupled with a risk-taking mentality may contribute to increased drowning risk among males when performing bystander rescues. While all people should be trained in safe rescue and resuscitation skills to improve outcomes and reduce risk [10], this can be extremely challenging in resource-poor environments. Turkish data indicate that males should be prioritized for such training, particularly those 15–34 years of age. Population-level coverage of rescue and resuscitation skills may be best achieved through school and workplace training programs. Training may also contribute to a reduction in incidents involving multiple fatalities of rescuers.

Of concern, 14% of all drowning fatalities of bystander rescuers in this study were of children and adolescents 5–14 years of age. Preventing drowning among this age group is challenging. While adult supervision is key for younger children, towards the upper end of this age group, adolescents may begin to recreate around water with peers, rather than parents or caregivers. It is, therefore, vital that this age group are taught non-contact rescue skills such as a ‘talk’, ‘throw’ or ‘reach’ rescue [33]. However, ‘throw’ and ‘reach’ rescues rely on access to safety equipment or tools that can be used in such rescues, which are often not available or are limited in Turkey, particularly at natural waterways [11].

In this study, natural fresh waters, such as streams, rivers and creeks were the most common location for fatal drownings of bystander rescuers. Rivers have been identified as a leading location for drowning, where prevention is often challenging due to changeable conditions and geographical isolation [34]. Swimming in rivers is often more risky than the beach or ocean, due to a lack of lifeguards and designated safe swimming areas [4]. While beach/sea was the second most common place for bystander rescue-related fatal drownings in this study, rescuers were significantly more likely to have been successful in rescuing the primary drowning victim at such locations. The specific circumstances of why this might be, such as the presence of others to assist, higher familiarity with conditions, and proximity to medical care, warrants further investigation.

In the current study, 33% of rescuers who died by drowning were successful in rescuing the primary drowning victim. This is an increase from 27% in a previous study conducted in Turkey between 2005 and 2008 [11]. However, such rates are significantly lower than the proportion of successful rescue attempts by bystanders reported in Australia [10]. This may be due to a stronger culture of swimming and lifesaving training, greater training in cardio-pulmonary resuscitation and the presence of public rescue equipment in Australia. However, in Turkey, attempts to rescue people who are drowning are often unsuccessful, and result in the deaths of both the primary drowning victim and the rescuer. Training in rescue and resuscitation, the installation of public rescue equipment at prominent drowning locations and enhanced public education are strategies that could be implemented and evaluated in Turkey to reduce the preventable loss of life in bystander rescue incidents. Similarly, resuscitation skills are likely to be a significant tertiary prevention skill, given that this study has identified that the vast majority (90%) of bystander rescuers died at the scene, rather than in the hospital. This indicates the need for rapid and effective on-site medical care [35].

With the development of technology, drones have started to be used in many areas, including for the prevention of drowning [36]. Using drones may offer the opportunity to reach submerged people earlier than traditional search strategies. The delivery of flotation devices to drowning victims with the help of drones may be an effective solution, especially in isolated locations or where the victim cannot be reached through traditional non-contact rescue methods [37]. However, this strategy relies on the victim being conscious and able to reach and use the floatation device.

This study utilized data collected from online media reports, due to a lack of data on drowning in Turkey. While media reports have been used to collate drowning data previously, both in Turkey [3,4,17] and elsewhere [18,19,20,21], the reliance on such data sources, highlights the need to strengthen drowning data surveillance systems in Turkey, a strategy recommended by the World Health Organization [24]. This includes disaggregation of external cause-code fatality data by injury mechanism [16]. Furthermore, investment in prevention would be well served by the establishment of a detailed drowning registry which nationally captures both fatal and non-fatal drowning incidents, to better identify at-risk groups and inform population-, location-and activity-based prevention efforts. The media-based reporting of drowning may be one strategy to collate data on drowning as it is in countries such as Australia [21], while also presenting an important opportunity for highlighting rescue safety when reporting such tragic incidents.

### Strengths and Limitations

The data presented in this study provide the prevalence of fatal drowning among bystander rescuers in Turkey. This is the most comprehensive study of rescuer drowning ever conducted in Turkey, with analyses identifying at risk groups, locations and activities on which to focus prevention efforts. However, there are several limitations associated with this study. As reported many times, there is a notable lack of data related to drowning in Turkey; therefore, media reports are used to identify cases of fatal drowning. While media reports have been found to have complete capture of bystander rescue-related drowning in Australia [21], this may not be the case in Turkey. Similarly, the variables reported in this study may not have been accurately reported by the media. Data on the race/ethnicity of those who drowned is not currently collected, nor is it well-reported within the media. It is, however, an important topic that is likely to impact drowning risk and, therefore, it is recommended that these data be collected, in so far as it is possible, in the future. This study did not capture a number of primary drowning victims. Fatal drowning rates are calculated per 100,000 population and, therefore, do not take into account exposure.

## 5. Conclusions

This study focused on the risk factors related to fatal drowning of bystanders during rescues in Turkey. Our study provides evidence that bystander rescuers are notably increasing with the number of drownings in Turkey. In the vast majority of fatal rescue attempts, bystander rescuers also failed to rescue the primary drowning victim. The results show that untrained bystander rescue attempts are not without risk and, thus, bystanders should be trained in non-contact recovery techniques. Consideration should also be given to population level education and to the exploration of emerging technologies.

## Figures and Tables

**Table 1:** Fatal drowning of bystander rescuers by status of rescue attempt, X^2^ (*p* value); Turkey, 2015–2019.

Factors	Total		Successful		Unsuccessful		X^2^ (*p* Value)
	N	%	N	%	N	%	
**Total**	237	100.0	77	32.5	160	67.5	-
**Sex**							
Male	213	89.9	70	90.9	143	89.4	0.134 (*p* = 0.821)
Female	24	10.1	7	9.1	17	10.6	
**Age group (years)**							
0–4	1	0.4	-	-	1	0.6	0.483 (*p* = 1.000)
5–14	34	14.3	7	9.1	27	16.9	2.563 (*p* = 0.118)
15–24	83	35.0	29	37.7	54	33.8	0.350 (*p* = 0.564)
25–34	49	20.7	16	20.8	33	20.6	0.001 (*p* = 1.000)
35–44	33	13.9	12	15.6	21	13.1	0.262 (*p* = 0.689)
45–54	22	9.3	10	13.0	12	7.5	1.859 (*p* = 0.231)
55+	15	6.3	3	3.9	12	7.5	1.139 (*p* = 0.397)
**Day of week**							
Weekdays	159	67.1	51	66.2	108	67.5	0.038 (*p* = 0.883)
Weekend	78	32.9	26	33.8	52	32.5	
**Place of death**							
At the scene	213	89.9	66	85.7	147	91.9	2.168 (*p* = 0.169)
Hospital	24	10.1	11	14.3	13	8.1	
**Season**							
Summer	151	63.7	59	76.6	92	57.5	**8.223 (*p* = 0.004)**
Spring	51	21.5	7	9.1	17	10.6	**4.916 (*p* = 0.029)**
Autumn	24	10.1	1	1.3	10	6.3	0.134 (*p* = 0.821)
Winter	11	4.6	10	13.0	41	25.6	2.879 (*p* = 0.109)
**Activity being undertaken prior to drowning**							
Swimming	69	29.1	34	44.2	35	21.9	**12.504 (*p* = 0.001)**
Non-water related recreation	55	23.2	11	14.3	44	27.5	**5.093 (*p* = 0.032)**
Having a picnic	44	18.6	12	15.6	32	20.0	0.670 (*p* = 0.478)
Occupational	31	13.1	9	11.7	22	13.8	0.194 (*p* = 0.837)
Recreational fishing	22	9.3	3	3.9	19	11.9	3.930 (*p* = 0.056)
Others	16	6.8	8	10.4	8	5.0	0.729 (*p* = 0.392)
**Location of drowning incident**							
Stream/River/Creek	81	34.1	20	26.0	61	38.2	3.412 (*p* = 0.079)
Beach/Sea	59	24.9	36	46.8	23	14.4	**29.147 (*p* < 0.001)**
Dam	30	12.7	6	7.8	24	15.0	2.443 (*p* = 0.146)
Lake/Pond	27	11.4	5	6.5	22	13.8	2.712 (*p* = 0.127)
Irrigation channel	26	11.0	8	10.4	18	11.3	0.039 (*p* = 1.000)
Pool	7	3.0	1	1.3	6	3.8	1.090 (*p* = 0.433)
Water hole	7	3.0	1	1.3	6	3.8	1.090 (*p* = 0.433)

Bold text indicates statistical significance.

**Table 2:** Fatal drowning of bystander rescuers by sex, X^2^ (*p* value), Turkey, 2015–2019.

Factors	Total		Male		Female		X^2^ (*p* Value)
	N	%	N	%	N	%	
**Total**	237	100.0	213	89.9	24	10.1	-
**Age group (years)**							
0–4	1	0.4	1	0.5	-	-	0.113 (*p* = 1.000)
5–14	34	14.3	28	13.1	6	25.0	2.467 (*p* = 0.127)
15–24	83	35.0	76	35.7	7	29.2	0.402 (*p* = 0.654)
25–34	49	20.7	46	21.6	3	12.5	1.088 (*p* = 0.427)
35–44	33	13.9	28	13.1	5	20.8	1.064 (*p* = 0.347)
45–54	22	9.3	20	9.4	2	8.3	0.029 (*p* = 1.000)
55+	15	6.3	14	6.6	1	4.2	0.211 (*p* = 1.000)
**Day of week**							
Weekdays	159	67.1	143	67.1	16	66.7	0.002 (*p* = 1.000)
Weekend	78	32.9	70	32.9	8	33.3	
**Place of death**							
At the scene	213	89.9	191	89.7	22	91.7	0.094 (*p* = 1.000)
In the hospital	24	10.1	22	10.3	2	8.3	
**Season**							
Summer	151	63.7	136	63.8	15	62.5	0.017 (*p* = 1.000)
Spring	51	21.5	45	21.1	6	25.0	0.192 (*p* = 0.610)
Autumn	24	10.1	22	10.3	2	8.3	0.094 (*p* = 1.000)
Winter	11	4.6	10	4.7	1	4.2	0.014 (*p* = 1.000)
**Activity being undertaken prior to drowning**							
Swimming	69	29.1	64	30.0	5	20.8	0.887 (*p* = 0.478)
Non-water related recreation	55	23.2	48	22.5	7	29.2	0.532 (*p* = 0.452)
Having a picnic	44	18.6	35	16.4	9	37.5	**6.333 (*p* = 0.023)**
Occupational	31	13.1	29	13.6	2	8.3	0.529 (*p* = 0.749)
Recreational fishing	22	9.3	22	10.3	-	-	2.733 (*p* = 0.141)
Others	16	6.8	15	7.0	1	4.2	0.146 (*p* = 1.000)
**Location of drowning incident**							
Stream/River/Creek	81	34.1	74	34.7	7	29.2	0.298 (*p* = 0.656)
Beach/Sea	59	24.9	54	25.4	5	20.8	0.236 (*p* = 0.805)
Dam	30	12.7	24	11.3	6	25.0	3.679 (*p* = 0.096)
Lake/Pond	27	11.4	25	11.7	2	8.3	0.248 (*p* = 1.000)
Irrigation channel	26	11.0	25	11.7	1	4.2	1.266 (*p* = 0.488)
Pool	7	3.0	6	2.8	1	4.2	0.137 (*p* = 0.531)
Water hole	7	3.0	5	2.3	2	8.3	2.696 (*p* = 0.151)

Bold text indicates statistical significance.

**Table 3:** Success of rescuers in rescuing and number of multiple drowning incidents.

Success of Rescuers in Rescuing	N	%
Unsuccessful	160	67.5
Successful	77	32.51
**Number of rescuer drowning fatalities per incident**	**N**	**%**
1	129	54.4
2	86	36.3
3	15	6.3
4	5	2.1
5	2	0.8

**Table 4:** Relative risk (95% confidence interval) by gender, age group, day of the week and season of drowning incident.

Factors	Number of Deaths	Drowning Rate	Relative Risk (95% Confidence Interval)
**Gender**			
Female	24	0.06	1
Male	213	0.52	8.81 (5.78–13.44)
**Age groups**			
0–4 years	1	0.02	1
5–14 years	34	0.54	34.64 (4.74–253.04)
15–24 years	83	1.28	82.21 (11.44–590.56)
25–34 years	49	0.78	49.80 (6.88–360.68)
35–44 years	33	0.54	34.76 (4.76–254.14)
45–54 years	22	0.46	29.20 (3.94–216.70)
55+ years	15	0.31	20.09 (2.65–152.10)
**Day of week**			
Weekend	78	0.10	1
Weekday	159	0.20	2.04 (1.56–2.67)
**Season**			
Winter	11	0.01	1
Autumn	24	0.03	2.18 (1.07–4.45)
Spring	51	0.06	4.64 (2.42–8.90)
Summer	151	0.19	13.73 (7.45–25.30)

## Data Availability

Data may be made available upon reasonable request. Please contact data custodian Ali Işın (isin_ali@hotmail.com) for further information.
